# Efficacy of Dietary Supplementation with *Capsicum Annum* L on Performance, Hematology, Blood Biochemistry and Hepatic Antioxidant Status of Growing Rabbits

**DOI:** 10.3390/ani10112045

**Published:** 2020-11-05

**Authors:** Hamada Elwan, Mostafa Abdelhakeam, Sally El-Shafei, Atef Abd El-Rahman, Zienhom Ismail, Abdalrahman Zanouny, Emad Shaker, Salim S. Al-Rejaie, Mohamed Mohany, Shaaban Elnesr

**Affiliations:** 1 Animal and Poultry Production Department, Faculty of Agriculture, Minia University, El-Minya 61519, Egypt; hamadaelwan83@mu.edu.eg (H.E.); mostafa.abdelhakem@mu.edu.eg (M.A.); Dr.zanouny@mu.edu.eg (A.Z.); 2 Department of Agricultural Chemistry, Faculty of Agriculture, Minia University, El-Minya 61519, Egypt; sally.ahmed@mu.edu.eg (S.E.-S.); atef.ahmed@mu.edu.eg (A.A.E.-R.); emad.shaker@mu.edu.eg (E.S.); 3 Animal Production Department, Faculty of Agriculture, South Valley University, Qena 83523, Egypt; z.shekhown@agr.svu.edu.eg; 4 Department of Pharmacology and Toxicology, College of Pharmacy, King Saud University, Riyadh 11451, Saudi Arabia; rejaie@ksu.edu.sa; 5 Poultry Production Department, Faculty of Agriculture, Fayoum University, Fayoum 63514, Egypt; ssn00@fayoum.edu.eg

**Keywords:** red hot pepper, rabbits, Mass detection, antioxidants, oxidative stress

## Abstract

**Simple Summary:**

The inclusion of phytochemicals in animal diets is a strategy that has been used to improve animal productivity by increasing the production yield. The addition of red-hot pepper (RHP) powder enhances productive rabbit performance and immunological, biochemical, and antioxidant statuses. Rabbits fed 2% RHP powder had higher weights. Thus, adding 1 or 2% RHP powder to rabbit diets is an easy, simple, and safe option for producers. The slaughter index and physical blood characteristics red blood cells (RBC’s), packed cell volume (PCV), hemoglobin (Hb), mean corpuscular volume (MCV), mean corpuscle hemoglobin (MCH) and mean corpuscle hemoglobin concentration (MCHC) were significantly improved by feeding growing rabbits on RHP levels. Low-density lipoprotein-cholesterol (LDL-C) and very-low were distinctly (VLDL-C) decreased (*p* < 0.05) when feeding rabbits either 1 or 2% RHP supplemented diets. Furthermore, supplementing the rabbit’s diet with 1% RHP led to a significant increase (*p* < 0.001) in the total antioxidant capacity when compared to the rabbits fed with the basal diet. Additionally, the thyroxin concentration was increased with RHP supplementation. A remarkably significant effect on serum and hepatic oxidative stress responses was observed with RHP supplementation.

**Abstract:**

Animals fed with a high amount of a wide range of antioxidants in their diet are significantly protected against oxidative stress. Powerful antioxidant substances such as vitamin E, vitamin C, and carotenoids are present naturally in red-hot pepper (RHP). This study hypothesized that using RHP may provide protection against oxidative stress and enhance animal physiological responses. Thus, this study aimed to investigate the effect of feeding New Zealand white rabbits with RHP-supplemented diets on their physiological and biochemical responses. New Zealand White rabbits (age = 6 weeks, n = 48) were split equally into three groups (n = 16 in each group). One group was fed a basal diet only (control group), with the other two groups fed a basal diet along with 1 and 2% RHP. Mass spectrometric analysis for the RHP methanolic extract showed some phenolic compounds, such as p-coumaric, sinapinic acids, vanillic, and luteolin, as well as catechin and its isomers. Hepatic antioxidant enzymes (SOD, GSH, GSH-Px, and CAT) were significantly elevated (*p* < 0.05) by feeding rabbits diets supplemented with 1 or 2% RHP. The addition of RHP significantly enhanced immune-responses; phagocytic activity, chemotaxis, TIg, IgG, IgM, and IgA increased when growing rabbits were fed RHP compared with the control group. In conclusion, dietary supplementation of 1 or 2% RHP may play a role as an enhancer of growth and immune response in growing rabbits.

## 1. Introduction

It is well-known that herbal supplements are used as feed additives to improve the health of humans and animals, due to their antioxidant properties and safety. Some of the most effective natural antioxidants, such as phenolic compounds, flavonoids, and phenylpropanoids, are found in natural feed additives. Red-hot pepper (*Capsicum annum* L.) is a widely used herb in animal nutrition. It is added in the range of 0.25 and 1.0% due to its essential role in decreasing accumulated lipid metabolites such as cholesterol in the body, which causes a decrease in the levels of triacylglycerols [1]. Red-hot pepper (RHP) is also rich in micronutrients, such as vitamins and minerals [2]. In addition, phytochemicals such as p-coumaric acid, catechin, vanillic acid, sinapic acid, and luteolin are present in *Capsicum annum*, and they exert many biochemical and pharmacological effects [3], antioxidant activities [4], and hypoglycemic effects [5]. A direct proportionality was found between antioxidant activity and total phenolic content in chili varieties [6].

The shortage in exogenous antioxidant resources may promote an unbalanced oxidative status, which may lead to cell deterioration, and thus, the consumption of natural antioxidants may offer a protective effect against reactive oxygen species (ROS) [7]. Miler et al. [8] reported that flavanones increased serum thyroid-stimulating hormones without altering thyroxin levels. Therefore, the addition of herbs, spices, and fruits to poultry feedstuff may be valuable for better performance. Under normal conditions, there is a homeostatic reaction between the generation of ROS and antioxidant defense actions used by animal cells to decrease ROS toxicity [9]. The family of superoxide dismutase (SOD) enzyme converts superoxide anions to hydrogen peroxide (H_2_O_2_), which can be directly destroyed by glutathione peroxidase (GSH-Px) or by catalase. Additionally, glutathione (GSH) is a major antioxidant that supplies decreasing equivalents for the GSH-Px-catalyzed reduction of H_2_O_2_ and lipid hydroperoxides.

Phenolic compounds play a crucial role in homeostatic animal antioxidant reactions; antioxidant activities (SOD, CAT, GSH-Px, and GSH) were significantly increased, and the lipid peroxidation markers were significantly decreased in diabetic rats exposed to 50 mg vanillic acid/ kg body weight [10]. Hot chili peppers have antidiabetic and antioxidant activities [11]. RHP, as a natural feed supplementation at different doses, can lead to inconsistent results, and the mechanisms of action are not well-understood [12]. We sought to study the mass qualitative scanning of RHP methanolic extract and the effect of RHP on growing a rabbit’s performance, hematology, blood biochemistry, and hepatic antioxidant activity.

## 2. Materials and Methods

This study was performed in the rabbit sector of the Experimental Farm, Faculty of Agriculture, Minia University, following the standard guidelines approved by the Animal and Poultry Production Department, Faculty of Agriculture, Minia University, Egypt. Research identification MU-FA-APPD/1/M/321/2018. Rabbits were housed individually in cages (40 × 50 × 35 cm) with an automated feeding and drinking system. Rabbits in all groups were housed and maintained under the same conditions. New Zealand white rabbits (age = 6 weeks, n = 48, 1 male: 1 female), with an average weight of 1081.10 ± 60.12 g, were randomly allocated into 3 groups (n = 16 in each group). The first group was fed the basal diet (the control), while the second and third groups were fed the basal diet supplemented with dried 1 or 2% RHP, respectively. All diets and water were supplied *ad libitum* throughout the experimental period (8 weeks).

### 2.1. RHP Preparation and Diet Formulations

RHP was purchased from a private and commercial market at El-Minya Governorate, Egypt. It was oven-dried at 50 °C to a constant weight. The dried RHP was finely milled, filtered (1 mm mesh), and placed in airtight bags at room temperature. Hot pepper powder was added and thoroughly mixed with other feed constituents for each batch at 0, 1, or 2%. There was a small amount of the basal diet blended with a respective volume of RHP as a small batch and then combined with a larger amount of the basal diet until properly mixed, then pelleted. A proper experimental diet formulation was chosen to meet the requirements of the National Research Council (NRC) [13].

### 2.2. RHP and Basal Diet Nutritional Analysis

Using the standard methods of the Association of Official Analytical Chemists (AOAC) [14], the nutritional composition of the basal diet and RHP was assessed where the methods numbers were as follows: (method 934.01) dry matter, (method 984.13) crude protein, (method 920.39) ether extract, (method 978.10) crude fiber and (method 942.05) ash. Using subtraction, the nitrogen-free content was calculated. The amino acid content of basal diet and RHP were duplicable, as determined by hydrolyzing the samples with 6 M HCl at 110 °C for 24 h according to Hong et al., [15] using high-pressure liquid chromatography analysis (HPLC) (Hitachi L-8900 Amino Acid Analyser, Tokyo, Japan) (Table 1).

### 2.3. Mass Scanning of Methanolic RHP Extract

**Figure 1:**
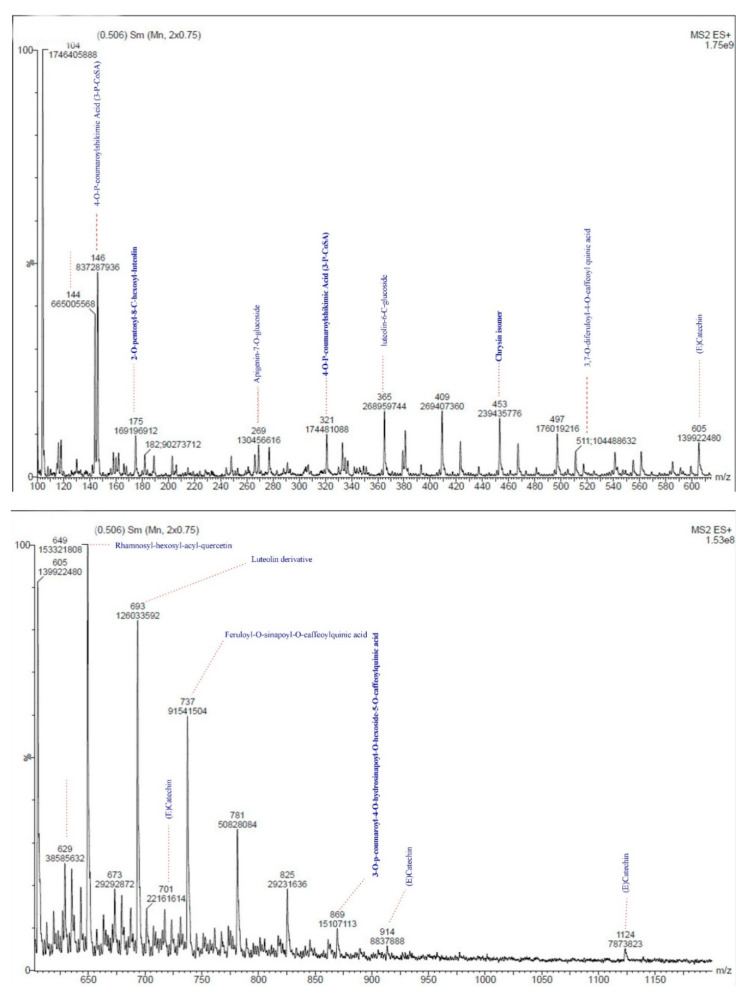
Mass spectrum of red-hot pepper methanolic extract in positive mode for LC-MS.

Briefly, the RHP sample was prepared for LC-MS analysis by dissolving 0.5 g RHP powder in 5 mL methanol at 5 °C three times every 24 h. Then the supernatant was concentrated on a rotary evaporator; 2 µL concentrated supernatant was injected directly into the LC-MS system. The full scan spectra (Figure 1) were carried out in the range 100–1150 m/z by using scan mode: profile; cycle time: 2 s; step size: 0.1 µL and pause between each scan: 2 min. The analysis was carried out using LC-MS AGERE, Giza, Egypt.

### 2.4. Slaughtering, Blood Sampling, and Hematological Studies

After 2 months, 10 rabbits from each group were weighed, slaughtered, and left to bleed completely. The slaughtered animals were deskinned and dressed out, and the hot carcass, including the head, was weighed and recorded. The edible offal (liver, heart, spleen, and kidneys) was separately weighed and recorded.

Blood was collected from the marginal ear vein between 07:00–08:00 a.m. in heparinized and non-heparinized tubes before slaughter. Blood samples in non-heparinized tubes centrifuged at 5000 rpm for 15 min, and the clear non-hemolyzed sera were separated and stored at −80 °C until used in the biochemical analysis. Red blood cells (RBCs), hemoglobin (Hb), packed cell volume (PCV), mean corpuscular volume (MCV), mean corpuscular hemoglobin (MCH), mean corpuscular hemoglobin concentration (MCHC), white blood cells (WBCs), and a granulocyte count were performed using a veterinary hematology analyzer (VetScan HM5 Hematology System, Abaxis UK).

### 2.5. Biochemical Measurements

#### Lipid Profile

Lipid metabolites in the serum were performed calorimetrically using kits from Vitro Scient. (Hanover, Germany). Triacylglycerols and total cholesterol concentrations were determined according to the protocol of commercial kits from Vitro Scient. (Hanover, Germany). HDL-cholesterol was assessed using the cholesterol E-Test Kit (Wako, Osaka, Japan). Serum LDL-C concentration calculated by the Friedewald equation, modified by Ahmadi et al., [16] as follows:

LDL (mg/dL) = TC/1.19 + TG/1.9 − HDL/1.1 − 38(1)

The very-low-density lipoprotein cholesterol (VLDL-C) concentration was calculated by the Friedewald equation as follows: VLDL-C = triacylglycerols /5.

### 2.6. Thyroid Hormones

Serum triiodothyronine (T3) and thyroxin (T4) levels were measured using commercial kits purchased from Immunotech Crop (Boston, MA, USA).

### 2.7. Total Antioxidant Capacity and Hepatic Oxidative Stress Biomarkers

Total antioxidant capacity (TAC) in the serum was determined using suitable commercial kits based on calorimetric methods (BioMed Chemical Company, Bader, Egypt). Liver homogenates were prepared by weighing 1 g of sample in 9 mL of ice-cold PBS (pH = 7.2), followed by homogenization using tissue homogenizer and centrifugation at 4 °C (10,000 rpm for 10 min). The supernatants were used to assay SOD, GSH, GSH-Px, malondialdehyde (MDA), and catalase (CAT) activity using commercial kits (BioMed Chemical Company, Bader, Egypt).

### 2.8. Immune Responses

The phagocytic activity was investigated according to the method described by Vetvicka et al. [17]. In brief, 0.1 mL of whole blood was added to 0.05 mL of diluted particle suspension (copolymers of 2-hydroxyethyl methacrylate) at 37 °C with agitation. After incubation (60 min) the suspension was smeared and stained using the Giemsa procedure. At least 200 leukocytes were evaluated.

Chemotactic movement and chemokinetic assays were conducted using a modified Boyden’s chamber described by Mohany et al. [18].

Total immunoglobulin levels were measured according to a method described by Mohany et al. [18]. Concisely, 208 mg ZnSO_4_.7H_2_O was dissolved in 1 L of freshly boiled distilled water. Then, 100 µL of the tested serum was mixed with 3 mL of ZnSO_4_ solution, shaken and incubated at room temperature for 1 h. Absorption caused by turbidity was measured photometrically at 660 nm. Serum IgG, IgM, and IgA concentrations were determined by commercial quantitative ELISA kits (GenWay Biotech Inc. San Diego, CA, USA).

### 2.9. Statistical Analysis

The one-way analysis of variance ANOVA was used to analyze the data, and separation of means was performed using Duncan’s multiple range tests using SAS software (Statistical Analysis System, version 9.1.3, World Headquarters, Cary, NC, USA). *p* < 0.05 level was considered significant.

## 3. Results

### 3.1. Mass Scanning of RHP

**Figure 2:**
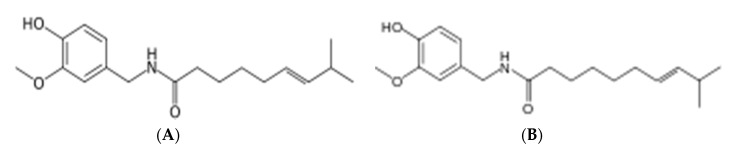
Mass scanning of red hot pepper methanolic extract, (**A**) capsaicin, and (**B**) homocapsaicin.

The mass spectrometric study of the RHP methanolic extract (Figure 1 and Figure 2) showed capsaicin (CPS) derivatives mainly as appearing in the mass spectrum library. The main fragment has been found in the spectrum as methoxybenzyl fragment after losing hydroxyl radical (m/z 104). For capsaicin: (6E)-N-[(4-Hydroxy-3-methoxyphenyl)methyl]-8-methylnon-6-enamide (CPS) with chemical formula C_18_H_27_NO_3_, the aromatic phenolic acid appeared on m/z 305. In LC-MS, adding Na is permitted in the procedure; the4-Hydroxy-3-methoxyphenyl fragment with the addition of Na in the positive mode shows m/z 146 fragments. Fission the aromatic fragment after the amide bond, dividing the CPS compound, giving m/z 182 fragments. CPS might lose methoxy and hydroxy groups from the aromatic ring, and that appears as m/z 269 +2H fragment. Homocapsaicin (HCPS) named as (6E)-N-[(4-Hydroxy-3-methoxyphenyl)methyl]-8-methylnon-6-enamide (m/z 319 + 2H), by losing the isopropyl fragment might show m/z 276 + H fragment. Fragment m/z 249 + H might refer to losing the isobutene unit or fragment from the homocapsaicin.

### 3.2. Growth Performance

It is worthy of note that all rabbits had 100% viability when fed diets supplemented with RHP or control diet during the experimental period. Body weight gain (BWG), average daily gain (ADG), average daily feed intake, and Feed/Gain ratio of the rabbits fed the experimental rations are shown in Table 2. The body weight gain (*p* = 0.003) and ADG (*p* = 0.015) of rabbits fed 1 or 2% RHP-supplemented diets were higher than rabbits in the control group. No significant (*p* > 0.05) difference in BWG was found between 1 or 2% groups. The mean daily feed intake was comparable (*p* > 0.05) among the experimental groups. In a similar trend, the Feed/Gain ratio was decreased (better) significantly (*p* = 0.032) in rabbits fed 1 or 2% RHP compared to those fed the control diet.

### 3.3. Carcass Characteristics

Mean slaughter weight, empty body weight and dressing percentage of the slaughtered rabbits fed experimental rations are shown in Table 3. The full digestive tract weight (*p* = 0.058), and total edible offal (giblets) weights (*p* = 0.058) heart (*p* = 0.078), liver (*p* = 0.125), kidneys (*p* = 0.078) and spleen (*p* = 0.095) were not significantly different among the experimental treatments. Furthermore, the pre-slaughter weight (*p* = 0.025), slaughter weight after bleeding (*p* = 0.018) the hot-carcass weight, including the head (HCW1) (*p* = 0.048) hot-carcass weight, including the head plus the total edible offal’s (HCW2) (*p* = 0.042), empty digestive tract weight (*p* = 0.002) and digestive tract length (*p* = 0.031), were significantly affected by increasing the RHP level from 0 to 2%. The corresponding values in rabbits fed 1% RHP were nearly the same as those fed 2% RHP.

### 3.4. Hematology

Data in Table 4 represent changes in hematological parameters at the end of the experiment (14 weeks of age). Total count of RBC’s, PCV, Hb, MCH, and MCHC were increased (*p* < 0.05) by feeding 1 or 2% RHP. Our results indicated that the addition of RHP to the diet had no significant effect (*p* > 0.05) on MCV. Regarding the total and differential counts of leucocytes, the addition of RHP to the diet led to an increase (*p* = 0.001) in the total count of WBCs compared with the control group. Moreover, the percentage of monocytes and neutrophils was increased (*p* = 0.001) with RHP supplementation. The rate of eosinophils and basophils was significantly decreased (*p* = 0.001) with RHP supplementation. Furthermore, the percent of lymphocytes did not differ (*p* > 0.05) in the RHP groups compared with the control group.

### 3.5. Cell-Mediated Immune Response

The cell-mediated immune response results illustrated in Table 4 revealed that rabbits fed RHP had higher (*p* = 0.001) phagocytic activity than the control group. Furthermore, the addition of RHP enhanced (*p* = 0.001) chemotactic movement, which was 33.30 and 35.89%, and no significant differences (*p* > 0.05) between RHP levels were observed. The chemokinase index data revealed that rabbits fed 1 or 2% RHP had higher values than the control group.

### 3.6. Humoral Immune Response

Regarding humoral immune response results, Table 4 illustrates that RHP addition led to increases (*p* = <0.001) in total immunoglobulins; moreover, 2% RHP resulted in the highest (*p* = 0.001) total immunoglobulins (Tig) when compared with the control or 1% RHP group. The IgG values had the same trend as TIg: 10.9, 12.0, and 11.6 while the percent of improvement (*p* = 0.001) was 9.17 and 8.25% for 1% RHP and 2% RHP, respectively. For IgM, rabbits fed RHP had higher (*p* = <0.001) IgM concentrations than the control group. However, there was no significant difference (*p* > 0.05) between RHP levels for IgM, and the IgA concentration had the same trend as IgM.

### 3.7. Thyroid Hormone Levels

The results of T3 and T4 are illustrated in Table 5. The addition of RHP to rabbit diets had no significant effect (*p* = 0.089) on T3 levels among all groups. In contrast, supplementing rabbit diets with RHP (1 or 2%) increased (*p* = 0.001) the T4 level compared to the control group. Thyroxin level was increased from 8.90 µg/dL for the control group to 11.8 and 14.2 µg/dL for 1 and 2% RHP, respectively.

### 3.8. Serum Lipid Profiles and TAC

Data regarding cholesterol, HDL-cholesterol, LDL-cholesterol, VLDL-cholesterol, triacylglycerols, and TAC are shown in Table 5. A significant decrease was recorded in cholesterol (*p* = 0.036), LDL-cholesterol (*p* = 0.032) and triacylglycerols (*p* = 0.031) in rabbits fed 1 or 2% RHP when compared to the control group. However, diets supplemented with 1 or 2% RHP enhanced (*p* = 0.041) HDL-cholesterol levels compared with the control group, whereas the VLDL-cholesterol level was decreased compared to the other groups (*p* = 0.028) when rabbits were fed 2% RHP. Regarding serum TAC, the data showed that the addition of RHP sharply increased (*p* = 0.001) serum TAC levels from 0.6 in the control rabbits to 0.9 and 1.2 in the rabbits 1% RHP or 2% RHP, respectively.

### 3.9. Hepatic Oxidative Stress and Antioxidants Biomarkers

The effects of RHP supplementation on hepatic oxidative stress biomarkers (SOD, GSH, GSH-Px, MDA, and CAT) of experimental rabbits are shown in Table 5. The GSH, GSH-Px, and SOD valuesin the liver tissues of RHP-supplemented groups (1 or 2%) were significantly (*p* < 0.05) increased compared to the control group. The SOD activity was extremely (*p* = 0.001) high in RHP-treated rabbits, where the rabbits fed on the control diet recorded 133.1 U/g tissue when rabbits fed on 1 or 2% RHP, (149.2 and 157.5 U/g tissue, respectively). GSH concentration was significantly increased (*p* = 0.001) in the RHP-treated groups compared to the control group. GSH-Px activity was significantly increased (*p* = 0.001) after treatment with RHP, with values of 141.7, 154.8 and 159.5 for the control, 1% and 2% RHP, respectively. Hepatic MDA levels were significantly suppressed by RHP supplementation (*p* = 0.020) compared to the control group. In contrast, hepatic CAT activity in the RHP groups was increased in the RHP-treated groups compared the control group.

## 4. Discussion

Alkaloids, terpenes, flavonoids, and glucosinolates are the most active phytochemicals found in plants [19]. However, each plant has a unique combination of these phytochemicals, and therefore their biological effects are expected to be different. Antioxidant activity is usually found in many phytochemicals, especially those rich in phenolic substances. Due to its synergistic effects, a mixture of phenolic sources may exhibit better outcomes than a single phenolic compound [6]. Our results show that RHP methanolic extract contains several phenolic substances, such as p-coumaric acid, catechin, vanillic acid, sinapic acid, luteolin, and the other firm peaks, which may be the phenolic glycosides or other compounds. All these compounds may have antioxidant properties, act as free radical scavengers, or be anti-infective agents [10]. Thus, the enhancement of a rabbit’s physiological and immunological status may be related to phenolic compounds of RHP. Our findings agreed with those of Sricharoen et al. [11], who reported that the phytochemical biological activities might be responsible for the functional effects of chili pepper.

Improving productive performance is one of the main goals in poultry and mammal production. Due to their high content of bioactive substances, medicinal plants as feed additives present a promising alternative to antibiotic growth promoters [20]. The addition of 1 or 2% RHP improved BWG, ADG, and Feed/Gain ratio, which may be due to the ability of RHP to improve the appetite and Feed/Gain ratio [21]. This led to a significant increase in final body weight (*p* < 0.002) and BWG (*p* < 0.003). The antioxidant activity of hot pepper phenolic compounds may be responsible for these positive changes. The positive effect of hot pepper has been reported in previous studies [3]. Additionally, capsaicin has been reported to stimulate pancreatic, intestinal activities, and increase bile acid secretion [22] and increase BWG in broiler chickens [1]. Carcass results could explain the role of active components in RHP. The highest carcass weight was recorded in the group fed with the highest percentage of RHP. This is easily explained, as rabbits in this group had better feed conversion ration than other groups, which resulted in heavier slaughter weight. Rabbits fed diets supplemented with hot pepper showed improvement in Feed/Gain ratio, which may be due to its stimulation, carminative, digestion, and antimicrobial properties [21]. Our findings agree with other scientific discoveries of Puvača et al. [1], who reported that supplementation of RHP could boost feed utilization by extending the small intestine surface area [21]. The improvement in the hot carcass weight and dressing % results from the reduction in the alimentary tract percentage, which may be due to the addition of RHP. This, in turn, increased the digestibility coefficient of nutrients while maintaining the acidic condition in the hindgut, which is optimized for better feed utilization [1].

Hematology is the professional responsible for the diagnosis and treatment of a wide variety of illnesses and diseases. According to Jain [23], Mitruka and Rawnsley, [24] and Zimmerman et al. [25], the normal range of hematological values of RBC’s, PCV, Hb, MCV, MCH, MCHC, WBC’s, neutrophils, lymphocytes, eosinophils, basophils, and monocytes of growing rabbits aged 1–3 months were 5.15–6.48 (n10^6^/µL), 38.1–44.1 (%), 10.7–13.9 (g/dL), 66.2–80.3 (fL), 19.5–22.7 (pg), 24.2–32.6 (%), 4.1–9.79 (n103/µL), 18.8–46.4 (%), 44.6–77.8 (%), 0–2.4 (%), 0.1–4.5 (%) and 0–13.1 (%), respectively. Our findings showed that all hematological parameters were within the normal range. The addition of different levels of hot pepper to rabbit diets increased the hematological parameters (RBCs, Hb%, PCV%, and erythrocyte indices) and had a direct effect on the blood characteristics. The biochemical results showed no harmful effects on blood components. The reduction of total blood cholesterol might be due to the active substrate of hot pepper supplementation such as P-coumaric acid, vanillic, sinapinic acid, luteolin, and catechin. This study demonstrated that supplementation of RHP in the rabbit diet could modulate lipid profiles by reducing TC and LDL-C, with HDL-C being increased. This result agrees with Manjunatha and Srinivasan [26], who found that the supplementation of 0.015% capsaicinoids into the diet could decrease serum TC, non-HDL-C, and TAG by 23, 44, and 14%, respectively. This effect might be caused by stimulating the conversion of cholesterol to bile acids, which, in turn, decreases cholesterol absorption and increases the excretion of fecal bile acid cholesterol [5]. Previous studies clarified that capsaicinoids exhibit cholesterol-lowering effects that could be achieved by the following mechanisms: inhibition of liver synthesis, induction of LDL-C uptake, acceleration of cholesterol degradation into bile acids, and an increase in bile acid excretion into the feces [4].

It is important to determine thyroid hormone activities and immune responses [27]. The addition of RHP (1 or 2%) to the growing rabbit diet enhanced thyroid function and immune responses, which reflected on rabbit growth performance. This result agrees with previous investigations of flavonoids, which have an impact on thyroid hormone synthesis and thyroid hormone metabolism [25]. In rabbits, the effect of phenolic compounds on thyroid function seems unclear; Chandra and De [28] reported that catechin could inhibit thyroperoxidase activity, decrease serum T3 and T4 levels, and elevate TSH concentrations. Thus, depending on the dose and time of treatment and species, flavonoids seem to affect the pituitary-thyroid axis [29] differentially. The conflicting results might be related to the type and source of the phenolic compounds, the dose, and the synergistic or antagonistic effects of the phenolic compounds.

Results of cell-mediated immune response assays revealed that phagocytosis and chemotactic movement were elevated by RHP supplementation; Warren et al., [30] reported that neutrophils have high levels of ascorbate, and this may be fundamental for their functional roles. Regarding humoral immune responses, our results indicated that rabbits fed dietary RHP (1 or 2%) had the highest values of TIg, IgG, IgM, and IgA. This could be related to the potent antioxidant constituents (p-coumaric, sinapinic acids, vanillic, luteolin, and catechin) of RHP supplementation. The protective effects of immune cells depend on cell membrane fluidity. The levels of polyunsaturated fatty acids in the cell membranes were increased, and the potential for membrane lipid peroxidation mediated by free radicals also increased [31]. Lipid peroxidation causes a decrease in membrane fluidity, which, in turn, affects immune responses [32].

Evaluation of the TAC offers more biologically relevant information than the individual levels of specific antioxidants of a given body fluid such as plasma or serum. The overall TAC influences the cumulative effect of all antioxidants present in the plasma and is used to evaluate the impact of several physiological responses [33]. Our results indicated that RHP supplementation significantly elevated the liver oxidative biomarkers SOD, GSH, GSH-Px, and CAT. These results agreed with those of Manjunatha and Srinivasan [26]. These effects could be attributed to the free radical scavenging activities of RHP, mainly phenols and flavonoids such as p-coumaric acid, catechin, vanillic acid, sinapic acid, and luteolin [34]. Finally, red pepper is rich in antioxidant compounds that have been linked to various health benefits [35]. In the current investigation, levels of TC, TAG, LDL-c, and VLDL-c were decreased, which led to the enhancement of physiological and productive responses in growing rabbits.

## 5. Conclusions

The present study suggests that the supplementation of a growing rabbit diet with RHP improves their growth performance, immunological response, biochemical, and hepatic antioxidant status. The most promising results were found in the group whose diet was supplemented with 1% RHP; no adverse effects were found in the group that received 2% RHP.

## Figures and Tables

**Table 1:** Ingredients, analyzed nutritional composition and amino acids of basal diet and red-hot pepper.

Items	Basal Diet	Red Hot Pepper
Ingredients (g/kg)		
Ground yellow corn	440	
Wheat bran	200	
Sugar beet pulp	200	
Soybean meal *	135	
Vegetable oil	10	
Limestone	5	
Sodium chloride	5	
Vitamin and mineral premix ^#^	5	
Analyzed nutritional composition (g/kg)		
Dry matter	937.8	922.9
Organic Matter	880.6	887.4
Crude protein	162.7	134.0
Crude fiber	102.4	67.2
Ether extract	30.1	53.3
Ash	57.2	35.5
Nitrogen free extract	585.4	632.9
Analyzed amino acids (g/100 g)		
Alanine	1.8547	1.3230
Arginine	0.9991	0.8890
Aspartic acid	0.8709	0.8980
Cysteine	0.2963	0.6130
Glutamic acid	0.6512	1.1130
Glycine	0.7257	1.4440
Histidine	0.4432	0.2460
Isoleucine	0.6802	0.8980
Leucine	1.2817	0.7980
Lysine	0.7216	0.6560
Methionine	0.3949	0.3790
Phenylalanine	0.7028	0.6490
Proline	0.7486	1.2320
Serine	0.7400	0.5130
Threonine	0.6598	0.3110
Tryptophan	0.2183	0.6770
Tyrosine	0.6739	0.2589
Valine	0.7355	1.0320

^#^ vitamin and mineral premix/kg contained the following IU/g for vitamins or minerals: A—4,000,000 IU/g; D3 5,000,000 IU/g; E—16.7 g; K—0.67 g; B1—0.67 g; B2—2 g; B6—0.67 g; B12—0.004 g; B5—16.7 g; pantothenic acid—6.67 g; biotin—0.07 g; folic acid—1.67 g; choline chloride—400 g; Zn—23.3 g; Mn—10 g; Fe—25 g; Cu—1.67 g; I—0.25 g; Se—0.033 g, and Mg—133.4 g (rabbit premix). * Soybean meal (44% crude protein).

**Table 2:** Growth performance of New Zealand white rabbits fed on dietary 0, 1 or 2% red hot pepper for 8 weeks of treatment (n= 16 rabbits per treatment).

Items	Treatments			SEM	*p*-Value
	Control	RHP 1%	RHP 2%		
Initial weight, (g)	1018.3	1086.6	1038.3	68.58	0.078
Final weight, (g)	2567.6 ^b^	2946.6 ^a^	2912.6 ^a^	108.68	0.002
Body weight gain, (g)	1549.3 ^b^	1860.0 ^a^	1874.3 ^a^	58.02	0.003
Average daily gain, (g)	27.6 ^b^	33.2 ^a^	33.4 ^a^	1.02	0.015
Average daily feed intake (g)	145	148	150	3.02	0.087
Feed/Gain ratio	5.2 ^b^	4.4 ^a^	4.4 ^a^	0.48	0.032

RHP = Red hot pepper; ^a, b^ values within a row with different superscripts differ significantly at *p* < 0.05.

**Table 3:** Rabbits carcass characteristics, dressing percentage, and giblets of New Zealand white as affected by dietary 0, 1, or 2 % red hot pepper for 8 weeks of treatment (n = 10 rabbits per treatment).

Items	Treatments			SEM	*p*-Value
	Control	RHP 1%	RHP 2%		
Pre-slaughter weight (g)	2450.6 ^b^	2865.6 ^a^	2893.3 ^a^	45.82	0.025
Slaughter weight after bleeding (g)	2288.6 ^b^	2702.4 ^a^	2727.8 ^a^	44.06	0.018
Hot carcass weight including head (HCW1) (g)	1346.7 ^b^	1833.5 ^a^	1853.4 ^a^	36.90	0.048
HCW1 + total edible offal’s (HCW2) (g)	1418.7 ^b^	1899.4 ^ab^	1920.1 ^a^	37.05	0.042
Dressing percentage					
HCW1/ EBW *	65.9 ^b^	74.3 ^a^	74.4 ^a^	0.53	0.049
HCW2/ EBW	70.2 ^b^	77.4 ^a^	78.3 ^a^	0.78	0.047
Digestive tract weight, (g)					
Full	399	405	410	11.02	0.058
Empty	152 ^b^	168 ^ab^	172 ^a^	5.80	0.002
Digestive tract length, (cm)	470 ^c^	518 ^b^	542 ^a^	8.90	0.031
Empty body weight, (g)	2041.6 ^b^	2465.4 ^a^	2489.8 ^a^	30.02	0.035
Edible offal’s (Giblets) weight, (g)					
Heart	5.4	5.0	5.1	0.15	0.078
Liver	52.0	47.6	47.5	4.87	0.125
Kidneys	13.0	11.7	12.6	1.45	0.078
Spleen	1.6	1.6	1.5	0.31	0.095
Total	72.0	65.9	66.7	5.02	0.058

RHP = Red hot pepper; * empty body weight; (EBW) = slaughter weight − digestive tract contents; ^a, b^ and ^c^ values within a row with different superscripts differ significantly at *p* < 0.05.

**Table 4:** Hematology, immunological responses, and thyroid hormone levels of New Zealand white rabbits fed on 0, 1, or 2% red hot pepper for 8 weeks of treatment (n = 10 rabbits per treatment).

Items	Treatments			SEM	*p*-Value
	Control	RHP 1%	RHP 2%		
Red blood cells (10^6^/mm^3^)	5.9 ^c^	6.3 ^b^	6.5 ^a^	0.04	0.045
Packed cell volume (%)	37.0 ^b^	39.0 ^a^	41.6 ^a^	0.92	0.035
Hemoglobin (g/100mL %)	11.3 ^b^	12.7 ^a^	13.6 ^a^	0.64	0.048
Mean corpuscular volume (fl)	62.5	61.9	63.4	2.83	0.091
Mean corpuscular hemoglobin (pg)	19.1 ^b^	20.1 ^a^	20.7 ^a^	0.09	0.012
Mean corpuscular hemoglobin concentration (g/dL)	30.6	32.6	32.7	0.50	0.057
White blood cells (10^3^/mm^3^)	5.4 ^b^	6.5 ^a^	6.4 ^a^	0.12	0.001
Lymphocytes (%)	62.2	61.4	61.2	0.96	0.340
Monocytes (%)	5.1 ^b^	5.4 ^a^	5.3 ^a^	0.05	0.001
Neutrophils (%)	30.5 ^b^	31.6 ^a^	32.0 ^a^	0.78	0.001
Eosinophils (%)	1.4 ^a^	1.2 ^b^	1.1 ^c^	0.03	0.001
Basophils (%)	0.8 ^a^	0.4 ^b^	0.4 ^b^	0.10	0.001
Cell-mediate immune responses					
Chemokinase index	3.9 ^b^	5.1 ^a^	5.2 ^a^	0.10	0.001
Phagocytic Activity (%)	38.7 ^b^	43.6 ^a^	43.7 ^a^	1.10	0.001
Chemotaxis (%)	43.2 ^b^	48.2 ^a^	48.3 ^a^	1.13	0.001
Humoral immune responses					
Total immunoglobulin (mg/dL)	11.8 ^b^	13.3 ^a^	13.4 ^a^	0.42	0.001
Immunoglobulin G (mg/dL)	10.9 ^c^	12.0 ^a^	11.6 ^b^	0.40	0.020
Immunoglobulin M (mg/dL)	0.36 ^b^	0.44 ^a^	0.47 ^a^	0.04	0.050
Immunoglobulin A (mg/dL)	0.53 ^b^	0.57 ^a^	0.58 ^a^	0.01	0.005
Thyroid hormones					
Triiodothyronine (ng/mL)	0.6	0.72	0.78	0.05	0.055
Thyroxin (µg/dL)	8.9 ^c^	11.8 ^b^	14.2 ^a^	0.63	0.035

RHP = Red hot pepper; ^a, b^ and ^c^ values within a row with different superscripts differ significantly at *p* < 0.05.

**Table 5:** Serum lipid profiles, total antioxidant capacity, and hepatic oxidative stress biomarkers of New Zealand white rabbits fed on 0, 1, or 2% red hot pepper for 8 weeks of treatments (n = 10 rabbits per treatment).

Items	Treatments			SEM	*p*-Value
	Control	RHP 1%	RHP 2%		
Cholesterol (mg/dL)	238.5 ^a^	156.5 ^c^	185.3 ^b^	0.98	0.036
HDL-Cholesterol (mg/dL)	94.9 ^c^	97.3 ^b^	105.3 ^a^	0.55	0.041
LDL-Cholesterol (mg/dL)	107.3 ^a^	25.1 ^c^	43.3 ^b^	0.59	0.032
VLDL-Cholesterol (mg/dL)	36.6 ^a^	36.2 ^a^	34.1 ^b^	0.18	0.028
Triacylglyceroles (mg/dL)	183.0 ^a^	181.1 ^b^	170.6 ^c^	0.92	0.031
Serum total antioxidant capacity (mM/L)	0.6 ^c^	0.9 ^b^	1.2 ^a^	0.05	0.001
Hepatic oxidative stress biomarkers					
Super oxide dismutase (U/g tissue)	133.1 ^c^	149.2 ^b^	157.5 ^a^	2.72	<0.01
Glutathione (mg/g tissue)	10.2 ^b^	12.0 ^a^	12.3 ^a^	0.43	0.001
Glutathione Peroxidase (U/g tissue)	141.7 ^b^	154.8 ^a^	159.5 ^a^	1.93	0.001
Malondialdehyde (nmol/g tissue)	12.4 ^a^	8.1 ^b^	7.2 ^c^	0.34	0.02
Catalase (U/g tissue)	31.1 ^c^	38.3 ^b^	42.0 ^a^	0.99	0.02

RHP = Red hot pepper; HDL-Cholesterol = high density lipoprotein cholesterol; LDL-Cholesterol = low density lipoprotein cholesterol; VLDL-Cholesterol = very low-density lipoprotein cholesterol. ^a, b^ and ^c^ Values within a row with different superscripts differ significantly at *p* < 0.05.
